# Core-, pan- and accessory genome analyses of *Clostridium neonatale*: insights into genetic diversity

**DOI:** 10.1099/mgen.0.000813

**Published:** 2022-05-13

**Authors:** Victoria Mesa, Marc Monot, Laurent Ferraris, Michel Popoff, Christelle Mazuet, Frederic Barbut, Johanne Delannoy, Bruno Dupuy, Marie-Jose Butel, Julio Aires

**Affiliations:** ^1^​ Université de Paris, UMR-S1139, F-75006, Paris, France; ^2^​ Plate-forme Technologique Biomics – Centre de Ressources et Recherches Technologiques, Institut Pasteur, F-75015, Paris, France; ^3^​ Institut Pasteur, Université de Paris, UMR-CNRS 2001, Laboratoire Pathogenèse des Bactéries Anaérobies, F-75015, Paris, France; ^4^​ Institut Pasteur, Université de Paris, Centre National de Référence des Bactéries anaérobies et Botulisme, F-75015, Paris, France; ^5^​ Assistance Publique-Hôpitaux de Paris, Hôpital saint Antoine, Infection Control Unit, F-75012, Paris, France

**Keywords:** *Clostridium neonatale*, closed genome, necrotizing enterocolitis, comparative genomics, pan-genome, core-genome

## Abstract

*
Clostridium neonatale* is a potential opportunistic pathogen recovered from faecal samples in cases of necrotizing enterocolitis (NEC), a gastrointestinal disease affecting preterm neonates. Although the *C. neonatale* species description and name validation were published in 2018, comparative genomics are lacking. In the present study, we provide the closed genome assembly of the *C. neonatale* ATCC BAA-265^T^ (=250.09) reference strain with a manually curated functional annotation of the coding sequences. Pan-, core- and accessory genome analyses were performed using the complete 250.09 genome (4.7 Mb), three new assemblies (4.6–5.6 Mb), and five publicly available draft genome assemblies (4.6–4.7 Mb). The *C. neonatale* pan-genome contains 6840 genes, while the core-genome has 3387 genes. Pan-genome analysis revealed an ‘open’ state and genomic diversity. The strain-specific gene families ranged from five to 742 genes. Multiple mobile genetic elements were predicted, including a total of 201 genomic islands, 13 insertion sequence families, one CRISPR-Cas type I-B system and 15 predicted intact prophage signatures. Primary virulence classes including offensive, defensive, regulation of virulence-associated genes and non-specific virulence factors were identified. The presence of a *tet*(W/N/W) gene encoding a tetracycline resistance ribosomal protection protein and a 23S rRNA methyltransferase *ermQ* gene were identified in two different strains. Together, our results revealed a genetic diversity and plasticity of *C. neonatale* genomes and provide a comprehensive view of this species genomic features, paving the way for the characterization of its biological capabilities.

## Data Summary

1. The four genomes assemblies used in the present study have been deposited in the European Nucleotide Archive database under BioProject PRJEB44145.
2. The closed genome assembly of *Clostridium neonatale* ATCC BAA-265^T^ (=LCDC 99A005^T^=CCUG 46077^T^) has been deposited in the European Nucleotide Archive database under accession no. ERS7257048 (GenBank accession no. SAMEA9534266).
3. The genome assembly of *C. neonatale* CB12 has been deposited in the European Nucleotide Archive database under accession no. ERS7257049 (GenBank accession no. SAMEA9534267).
4. The genome assembly of *C. neonatale* CC3_PB has been deposited in the European Nucleotide Archive database under accession no. ERS7257050 (GenBank accession no. SAMEA9534268).
5. The genome assembly of *C. neonatale* LF22 has been deposited in the European Nucleotide Archive database under accession no. ERS7257051 (GenBank accession no. SAMEA9534269).

All *C. neonatale* genome sequences utilized for the comparative genome analysis are listed in Table 1.

**Table 1.** *
C. neonatale* strains and genome data NEC+, preterm infants with a diagnosis of necrotizing enterocolitis (NEC).

* C. neonatale * strain ID	Date of isolation (reference)	Sample type	Sequencing technology	Contigs/genome close level	Contig N50 (bp)	Contig L50	Genome size (kb)	G+C (%)	CDS	tRNAs	rRNAs	Repeat regions	Accession no.
250.09 *, †	2002 [8]	Stool/NEC+	Pacbio HiSeq	1 Closed	4 753 394	1	4753	28.61	4356	85	33	275	ERS7257048 (This study)
CC3_PB†	2015 [10]	Stool/NEC+	Pacbio HiSeq	6 Contigs	5 137 519	1	5675	28.60	5505	91	32	37	ERS7257050 (This study)
LF22†	2006	Stool	HiSeq	288 Contigs	55 509	32	5598	28.67	5602	78	11	53	ERS7257051 (This study)
CB12†	2015 [10]	Stool/NEC+	HiSeq	83 Contigs	119 741	14	4612	28.38	4259	74	11	24	ERS7257049 (This study)
NEC25	2011 [9]	Stool/NEC+	MiSeq	3‡ Contigs	2 546 558	1	4739	28.67	3932	77	28	221	PRJEB26968
NEC26	2012 [9]	Stool/NEC+	MiSeq	3‡ Contigs	2 546 622	1	4738	28.86	3808	77	28	218	PRJEB26973
NEC32	2012 [9]	Stool/NEC+	MiSeq	3‡ Contigs	2 546 635	1	4738	28.75	3891	77	28	220	PRJEB27003
NEC86	2010 [9]	Stool/NEC+	MiSeq	3‡ Contigs	2 546 783	1	4739	28.58	4304	77	28	230	PRJEB26949
C25	2012 [9]	Stool samples	MiSeq	3‡ Contigs	2 546 563	1	4739	28.64	3937	77	28	228	PRJEB26947

*Reference strain ATCC BAA-265^T^.

†Strains were isolated from independent individuals in different spatiotemporal settings.

‡Contigs containing ‘N’ stretches.

NEC+, satrin isolated from a NEC case.

**Impact Statement**

We provide the whole genome assembly of *Clostridium neonatale *, a recently validated species classified within the genus *Clostridium sensu stricto.* The *C. neonatale* genome was manually curated and used to perform comparative genomics of this potential opportunistic pathogen associated with necrotizing enterocolitis, a gastrointestinal disease affecting preterm neonates. The *C. neonatale* genome is characterized by an open pan-genome state with genetic diversity and a flexible gene repertoire. Our data provide insights into this microorganism's biological capabilities, plasticity and defence mechanisms, and highlight the need for further extending the number of *C. neonatale* sequenced genomes to elucidate the pathogenic potential of this species.

## Introduction

The bacterial genus *Clostridium* was first proposed by Prazmowski in 1880 [1] and its description was recently amended proposing that members of the genus *Clostridium* Prazmowski be restricted to cluster I *sensu stricto* species [2]. The genus *Clostridium* encompasses more than 336 species and 12 subspecies [3] and contains Gram-stain-positive, spore-forming, obligate anaerobes. *Clostridium* species are found in the environment, and are common inhabitants of the intestinal microbiota of humans and animals. In humans, some *Clostridium* species are associated with severe diseases in adults and children such as botulism, tetanus, gangrene, antibiotic-associated diarrhoea and food poisoning [4]. Concerning non-pathogenic *Clostridium* species, they possess properties allowing them to occupy specialized niches thanks to specific biocatalytic capabilities that may be exploited for the production of chemical substances through biological transformations. Indeed, different *Clostridium* species are studied as possible cell factories for the production of chemicals through processes offering an alternative to petrochemical routes [5].

Necrotizing enterocolitis (NEC) is one of the most common neonatal intestinal diseases occurring in extremely preterm neonates [6]. The incidence of NEC ranges from 3 to 15 % in high-income countries and is associated with a high mortality rate and long-term complications [6]. In 2002, *Clostridium neonatale* was isolated for the first time from blood cultures of a premature neonate during an outbreak of NEC [7]. In 2014, Bouvet *et al*. proposed to classify *C. neonatale* as a novel species within the genus *Clostridium sensu stricto* (cluster I) [8]. In 2018, *C. neonatale* sp. nov. was validated [9]. The closest related species to *C. neonatale* are *Clostridium beijerinckii* and *Clostridium butyricum* [8, 9]. Between 2002 and 2018, the confusing status of this species explains the absence of data on its isolation, identification and clinical significance. Consequently, misidentification and/or underrepresentation of *C. neonatale* populations during previous studies may have occurred. Nevertheless, few studies have isolated and identified *C. neonatale* strains from neonatal stools [7, 8, 10], suggesting that *C. neonatale* is part of the infant gut microbiota. More recently, *C. neonatale* was again associated with NEC in both a multicentre [11] and a regional study [12]. Interestingly, *C. butyricum* has been also proposed to participate in the onset of NEC [11, 13]. At the bacterial level, no specific virulence factors have been found associated in either *C. neonatale* or *C. butyricum *. Although a putative haemolysin-like protein in *C. butyricum* strains was reported [13], the involvement of this toxin in the pathophysiology of NEC needs to be demonstrated. Additionally, in animal models of NEC, *C. neonatale* was significantly overrepresented in colonic mucosa samples from preterm piglets with NEC [14] and *C. butyricum* was shown to produce intestinal NEC-like lesions in chickens and quails [15–18]. Although clinical and epidemiological data have associated *C. neonatale* with NEC [7, 10–12], the underlying mechanisms of this species’ pathogenicity are unknown.

Two draft genomes of the reference strain *C. neonatale* ATCC BAA-265^T^ were proposed in 2016 and 2018 [9, 19], but to date, very little is known about *C. neonatale* population structure, evolution and genetic characteristics. In the present study, to investigate *C. neonatale* genomics and determine its genomic diversity, we generated a closed genome of the reference strain ATCC BAA-265^T^ and performed a manual expert curated annotation of all coding sequences (CDS). We also generated three new draft genomes of *C. neonatale* clinical isolates (CB12, CC3_PB and LF22) and included five publicly available genomes to compare variability in pan-genome, core-genome, unique and accessory genes. A comparative genome analysis was performed to identify *C. neonatale* genomic features, characterize potential defence mechanisms, mobile genetic elements, virulence genes and antibiotic resistance genetic determinants potentially involved in the pathogenicity of *C. neonatale *.

## Methods

### Strains, media and growth conditions

*C. neonatale* strains CB12, CC3_PB and LF22 included in the present study belong to our laboratory collection and were previously isolated from stool samples in different spatiotemporal settings (Table 1) [11, 20]. The reference strain ATCC BAA-265^T^, previously named strain 250.09 [8], was also included. For *C. neonatale* growth, liquid TGYH broth (per litre: tryptone 30 g, glucose 5 g, yeast extract 20 g and haemin 5 mg) or TGYH agar media were used and bacteria were incubated for 48 h at 37 °C under anaerobic conditions (CO_2_/H_2_/N_2_, 10 : 10 : 80; anaerobic chamber MACS; bioMérieux).

### Whole genome sequencing, contig assembly and annotation

DNA extraction was performed on 24 h bacterial liquid cultures after centrifugation using the DNA easy UltraClean microbial kit (Qiagen). Four *C. neonatale* genomes (250.09, CC3_PB, LF22 and CB12) were generated in this study (Table 1). Paired-end libraries were prepared according to the manufacturer’s instructions using the TruSeq DNA PCR-free sample prep kit (Illumina) and sequenced on a HiSeq 2500 system (Illumina). Genome sequences were assembled *de novo* using SPAdes 3.14.1 [21], combining information from 51 to 89 *k-mer* sizes. Scaffolding and gap filling were performed using SSPACE and GapFiller. Additionally, the *C. neonatale* 250.09 and CC3_PB genomes were sequenced using PacBio single-molecule real-time technology and sequences were assembled using the SMRT Analysis v2.3.0 software and the Hierarchical Genome Assembly Process (HGAP_3) workflow. For both strains 250.09 and CC3_PB, a genome hybrid assembly based on the PacBio and HiSeq sequences was obtained using SPAdes 3.14.1. The reference strain 250.09 genome assembly was ordered against the closest related species with an available complete genome, *C. beijerinckii* NCBIM 8052 (accession no. NC_009617.1). The reference strain 250.09 genome was subsequently used for ordering the other draft genome assemblies. All genome assemblies were annotated automatically using the MicroScope pipeline platform v3.14.3 [22]. The *C. neonatale* 250.09 reference strain genome automatic annotation was manually curated and each CDS function was verified and amended when necessary using the Microscope platform tools [22] and as described by Monot *et al*. [23].

### Comparative genomic analysis

The genetic diversity among the genomes was analysed using the average nucleotide identity (ANI) based on ANIm (MUMmer) by applying the Jspecies web server (last accessed March 2021) [24] and the resulting matrix was clustered and visualized using R package heatmap software [25]. Genome visualization of the coding regions was compared using the CGViewer web server (last accessed July 2021) [26]. The assignment of each CDS in the *C. neonatale* 250.09 genome was transferred to the other genomes included in this study using the MicroScope pipeline (sequence values of similarity and identity of 80 %). The pan- and core-genome analyses were performed using the Bacterial Pan Genome Analysis BPGA pipeline (v.1.3) [27]: sequence data were pre-processed and clustered to generate orthologous proteins using the USEARCH algorithm (settings: 80 % cut-off) [28]. The median sizes of the pan-genome data were generated using Heap's mathematical model, *n* = k*N ^γ^, where *n* is the total number of all non-redundant gene families in the pan-genome, *N* is the number of genomes, and both ‘k’ and ‘γ’ are proportionally and exponent constants [29]. The curve fitting of shared genomes was performed using the formula for least-squares fitting of the exponential regression decay represented by *n*=ke *n*/τ+*tg*θ, where *n* is the number of genes, *N* is the number of genomes, ‘e’ is the Euler number, and ‘k’, ‘τ’ and *tg*θ are constants definite to fit the formula [29].

The OrthoVenn2 web server (last accessed July 2021) with default parameters (E-value 1×10^−5^, inflation value 1.5) was used to identify orthologous gene clusters in the assembled genomes [30].

Metabolism capabilities were predicted using respectively the MicroCyc and the antiSMASH (V5.0.0) tools included in the MicroScope pipeline. Potential homologous genes associated with virulence were identified using the VFDB database default parameters (blast settings E-value <1×10^−5^ and an additional cut-off of 75 % minimum alignment) [31]. The Resistance Gene Identifier software of the Comprehensive Antibiotic Resistance Database web server (last accessed June 2021) was used for identification of potential homologous genes associated with antibiotic resistance [32].

### Identification of insertion elements, genomic islands, CRISPR-Cas systems, prophages and plasmid elements

The presence of genomic islands (GIs) was predicted by integrating the IslandPath-DIMOB and SIGI-HMM methods using the IslandViewer 4 database (last accessed June 2021) [33]. Insertion sequences (ISs) were predicted using the ISFinder database (E-values <0.01) (last accessed June 2021) [34]. Putative CRISPR-Cas systems were identified using the CRISPRCasFinder online tool with default parameters (last accessed June 2021) [35]. Bacteriophage sequences were identified using the PHAge Search Tool Enhanced Release web server (last accessed June 2021) [36]. Identification of plasmid elements was performed using PlasClass [37] which assigns probabilities to sequences that are classified as having a plasmid origin if the probability that it belongs to a plasmid class is >0.5.

### Antibiotic susceptibility

Antimicrobial susceptibility testing of strains was performed using the disc diffusion method as previously described [20] and according to the EUCAST recommendations [38]. Antibiotics (bioMérieux) tested were as follows: amoxicillin, amoxicillin-clavulanic acid, piperacillin, piperacillin-tazobactam, ertapenem, imipenem, cefoxitin, tetracycline, tigecycline, chloramphenicol, moxifloxacin, metronidazole, linezolid and vancomycin. Minimum inhibitory concentrations (MICs) for tetracycline, clindamycin and cefotaxime were determined using the E-test strips as specified by the manufacturer (bioMérieux). Antimicrobial susceptibility testing was performed twice.

## Results

### *C. neonatale* 250.09 closed genome characteristics

**Fig. 1.**
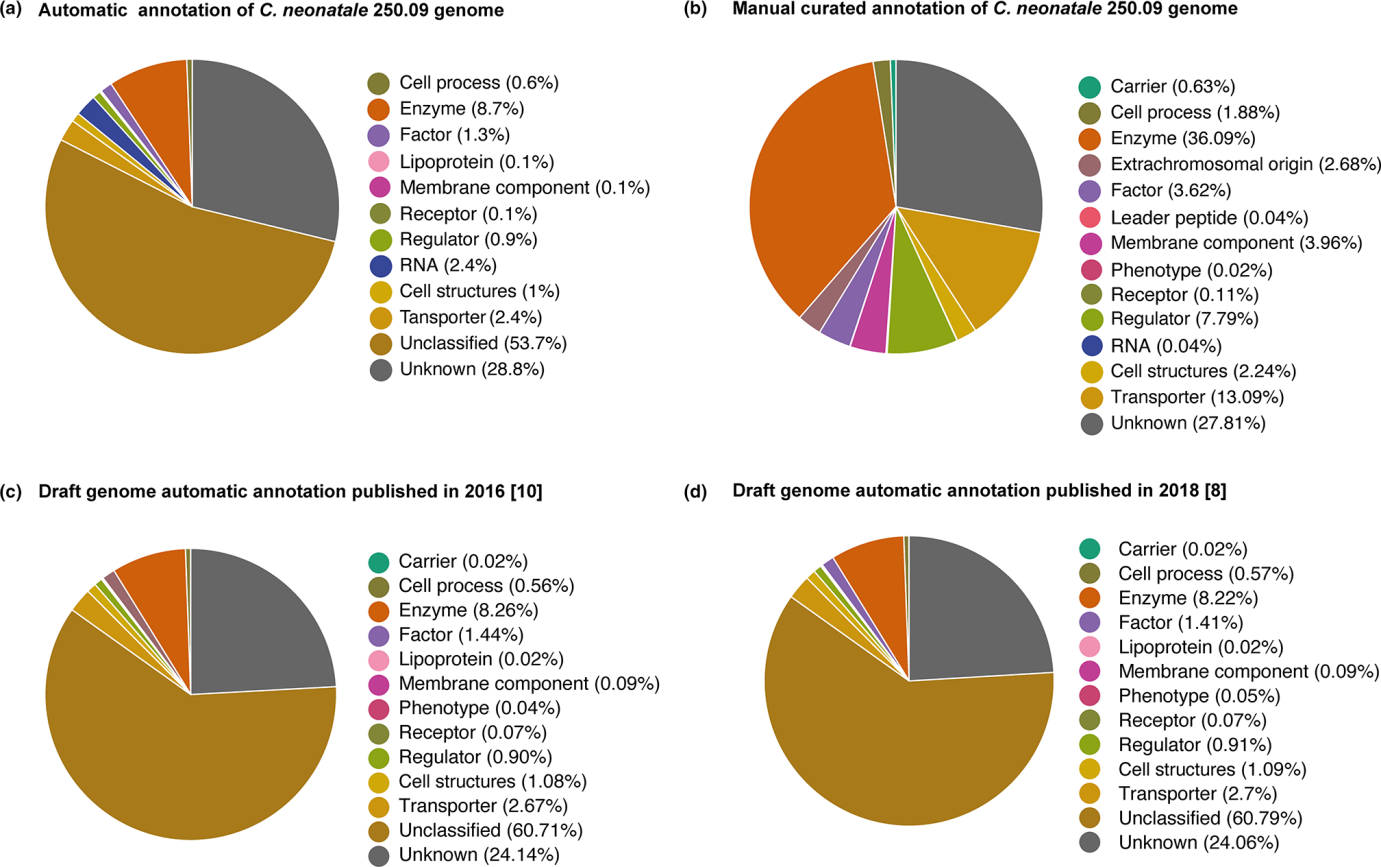
Pie chart comparing the automatic and the manually curated annotation of the *C. neonatale* 250.09 genome. (**a**) Automatic annotation, this study. (**b**) Manual curated annotation, this study. (**c**) Draft genome automatic annotation published in 2016 [11]. (**d**) Draft genome automatic annotation published in 2018 [8].

The *de novo* genome hybrid assembly based on the PacBio and HiSeq sequences resulted in one contig corresponding to the closed assembled chromosome of *C. neonatale* reference strain 250.09 of 4 753 394 bp, with a GC content of 28.6 %, 33 rRNA operon copies, 85 tRNA genes and 4356 CDS. Manual expert curation and functional annotation of each automatically assigned CDS contributed to reduce CDS numbers by 5 % (from 4588 to 4356) and remarkably increased the annotated genes from 11 to 44 %. In particular, we observed a higher percentage of identified genes related to enzymes (8–36 %), cell process (0.6–2 %), factor components (1–4 %), regulators (0.9 %–8 %), cell structures (1–2 %) and transporters (2–13 %) (Fig. 1). The manual curation and CDS verification also allowed us to annotate new gene categories belonging to carriers, extrachromosomal origin elements and membrane components (Fig. 1).

### Features of the *C. neonatale* genomes

**Fig. 2.**
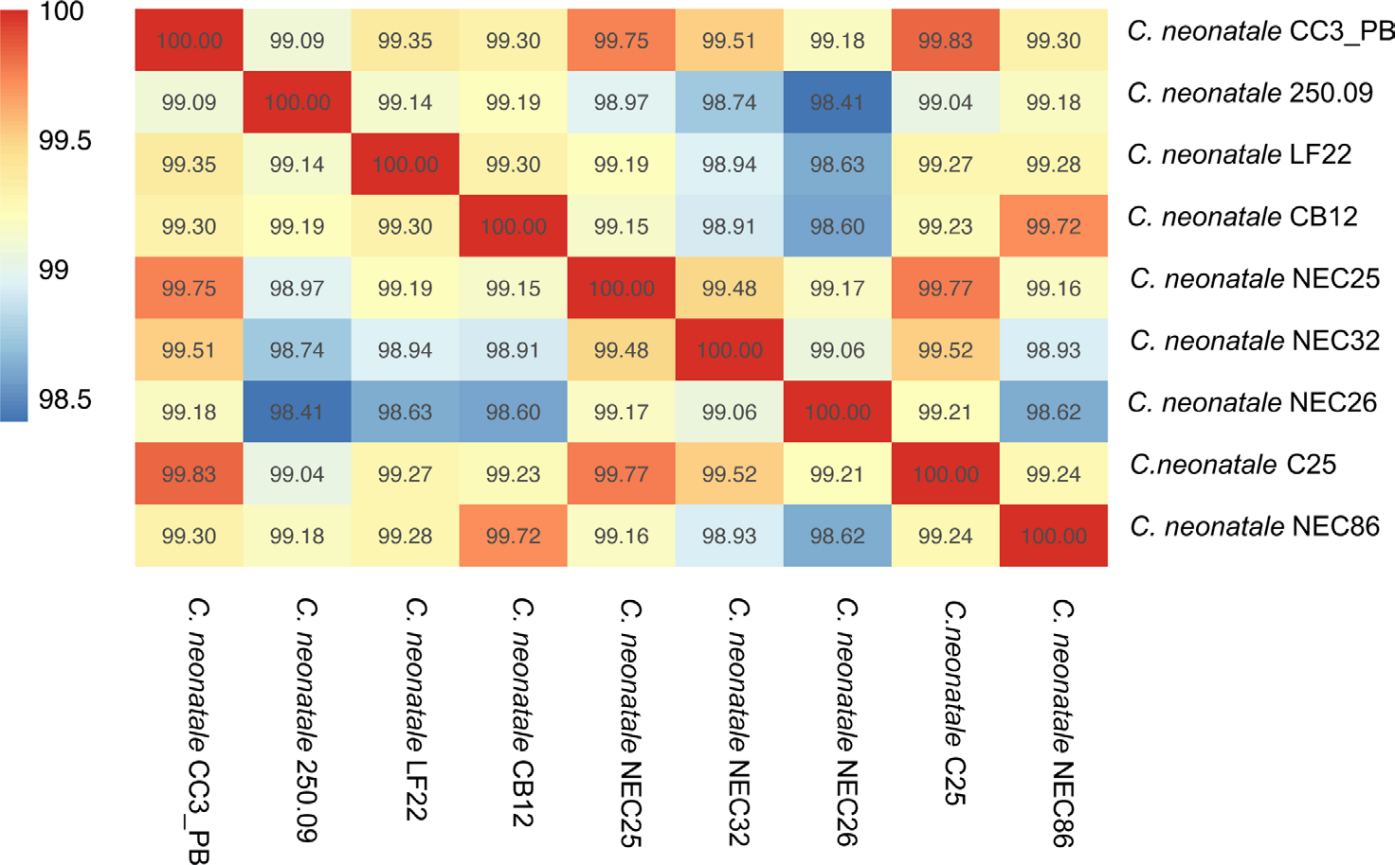
Heatmap representing the degree of genome similarity based on ANI. The heatmap was derived from the high similarity (dark red) and low similarity (blue) of CDS derived from the nine *C *. *neonatale* genomes.

**Fig. 3.**
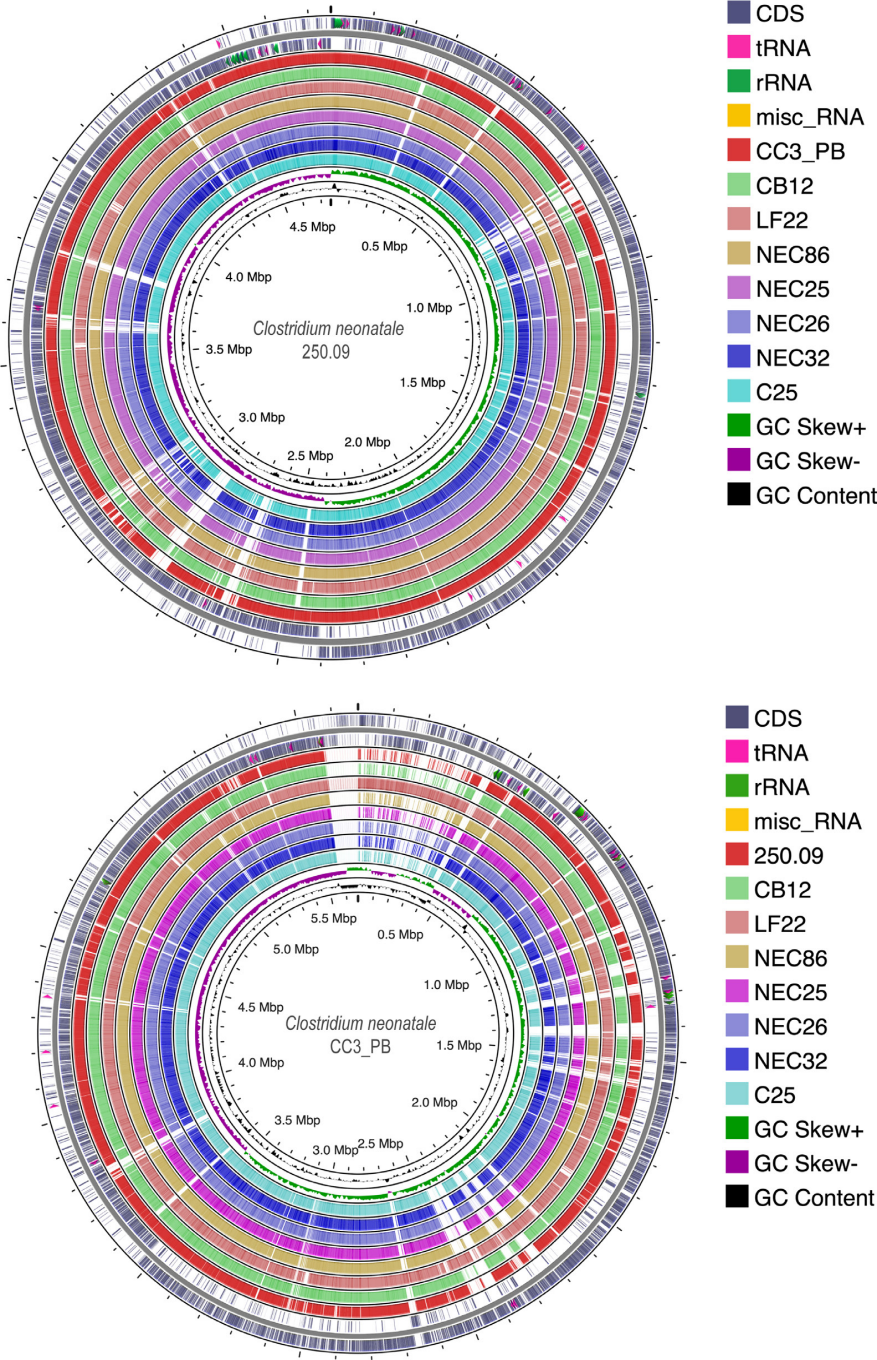
*
C. neonatale* circular genome comparison maps. The upper and lower figures show the blast comparison between genomes and 250.09 or CC3_PB as the reference genomes, respectively. Genome scale (Mbp) is indicated on the innermost circles. Origin of replication is positioned at 0 Mbp. Regions in white correspond to variable areas between genomes. The two outermost circles indicate locations of gene coding regions in the plus (circle 1) and minus (circle 2) strands. Rings 3–10 indicate blast comparison with the contigs of the different strain genomes. Circle 11 shows G+C skew in the plus (green) and minus (purple) strands. The innermost rings indicate G+C content (deviation from average).

The genomes of *C. neonatale* 250.09, CC3_PB, LF22 and CB12, and five publicly available genomes were used for comparative genomic analysis. Their G+C content ranged from 28.38% (CB12) to 28.86 % (NEC26). Genome length ranged from 4.6 Mb (CB12) to 5.6 Mb (CC3_PB) with a number of predicted protein coding genes of 4259 and 5505, respectively (Table 1). Whole-genome pairwise sequence comparisons showed an ANI of 98.41 % for the most distant strains (250.09 and NEC26), while the closest strains showed an ANI of 99.83 % (CC3_PB and C25) (Fig. 2). Whole-genome circular comparative maps revealed differences in the genomic regions: the CC3_PB genome showed the greatest differences compared to the other genomes (Fig. 3).

### Pan- and core-genome analyses of *C. neonatale*

**Fig. 4.**
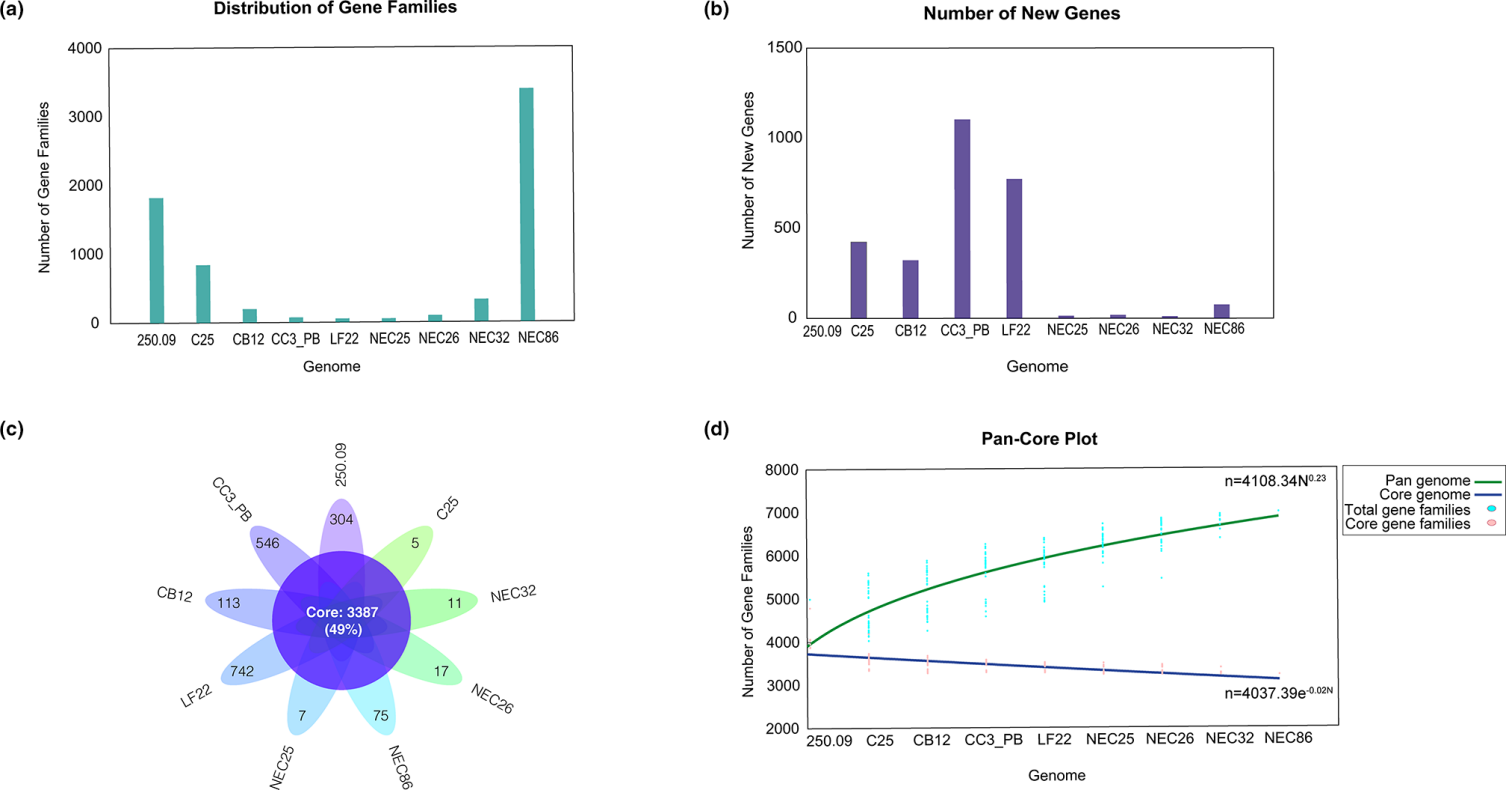
Pan- and core-genome analysis of *C. neonatale* strains. (**a**) Distribution frequency of the gene families within genomes. (**b**) Number of new genes added to each genome. (**c**) Flowerplot illustrating the core-genome size (flower centre), and unique genes for each strain (flower petals). (**d**) Cumulative curves showing the downward trend of the core gene families (in blue) and the upward trend of the pan gene families (in green) with the increase in the number of genomes. Power and exponential equations are noted.

The distribution of gene families and new genes within the pan-genome are shown in Fig. 4(a, b). A total repertoire of 6840 genes was identified in the pan-genome and 408 (5.9 %) to 1190 (17 %) genes were part of the accessory genome (Table S1, available in the online version of this article). We observed from five to 742 unique genes among strain genomes (strain C25 and LF22, respectively) (Fig. 4c). The *C. neonatale* NEC86 genome had the highest number of gene families (*n*=3400) and strain CC3_PB had the highest number of new genes (*n*=1103). The largest genomes (CC3_PB and LF22) had the highest number of unique genes (Fig. 4c). The number of exclusively absent genes ranged from one to 224 (strains C25 and 250.09, respectively) (Table S1).

The fitted curve of the pan-genome was determined using the power-law regression based on Heap’s law [28]: for *N* (number of genomes)=9, the resultant pan-genome fitted model is 4108.34×*N* ^0.23^ with a power-law coefficient γ of 0.23. This indicates an open pan-genome indicating the addition of accessory genes with each new genome introduction (Fig. 4d). When extrapolating to *n*=5000 genomes, it is expected that sequencing another one would add one gene to the pan-genome.

Core-genome analysis converged to a gene subset of 3387 and the number of shared genes decreased with new genome introduction. The core-genome development tend to plateau, as confirmed by the least-squares fitting of exponential regression decay to the mean values model, 4037.39e^-0.02*N^ (Fig. 4d).

**Fig. 5.**
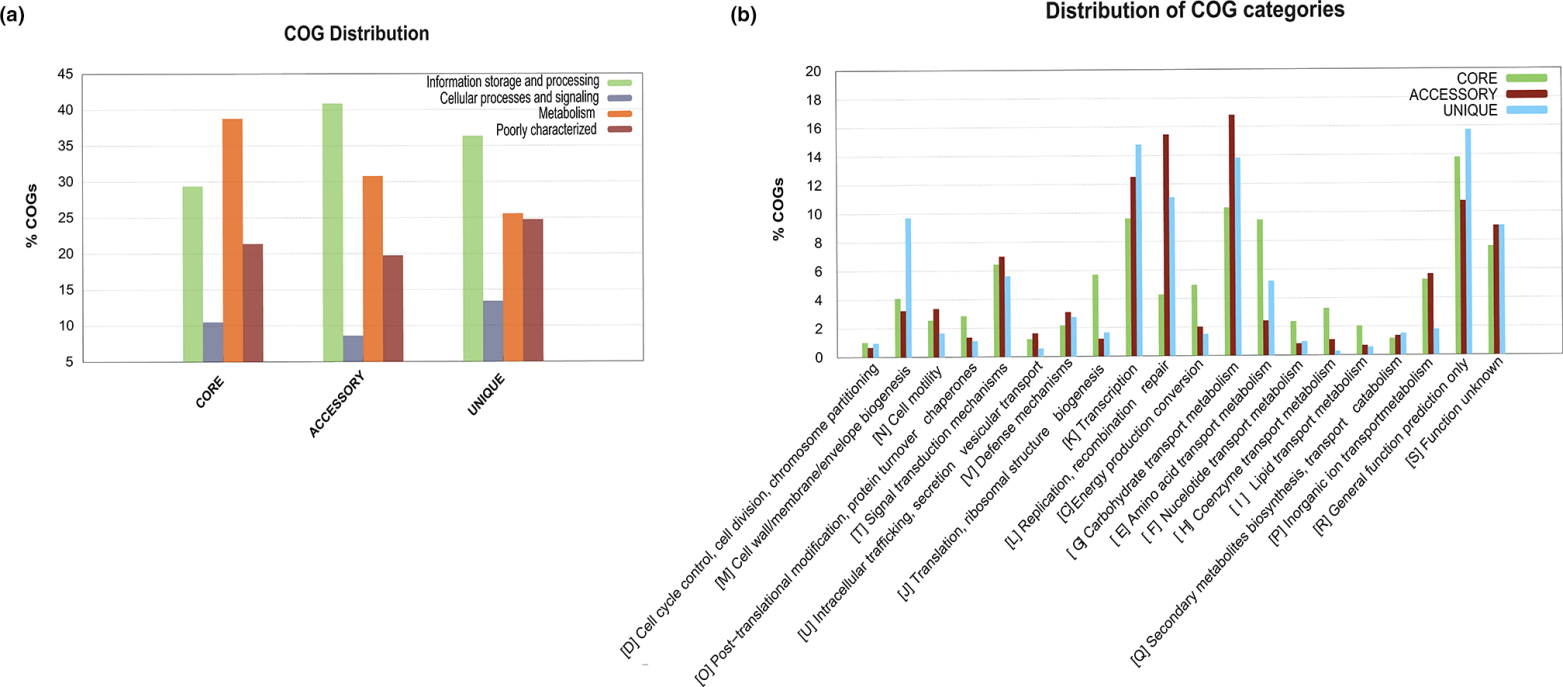
Functional categories of genes. (**a**) COG distribution. (**b**) COG distribution categories for the core, accessory and unique genome.

The distribution of the functional annotation genes among COG categories is presented in Fig. 5. The four major COG categories were related to ‘metabolism’ (25–38 %), ‘cellular processes and signalling’ (8–13 %), ‘information storage and processing’ (29–40 %), and ‘poorly/unknown functions’ (20–25 %) (Fig. 5a). The core gene families was enriched in ‘amino acid transport and metabolism’ (9 %) and ‘general function prediction’ (13 %) (Fig. 5b). Categories such as ‘replication, recombination and repair’ (15 %) and ‘carbohydrate transport and metabolism’ (16 %) were more represented in the accessory gene families. ‘Cell wall/membrane/envelope biogenesis’ (9 %) and ‘general function prediction’ categories (15 %) were enriched in the unique gene families (Fig. 5b).

**Fig. 6.**
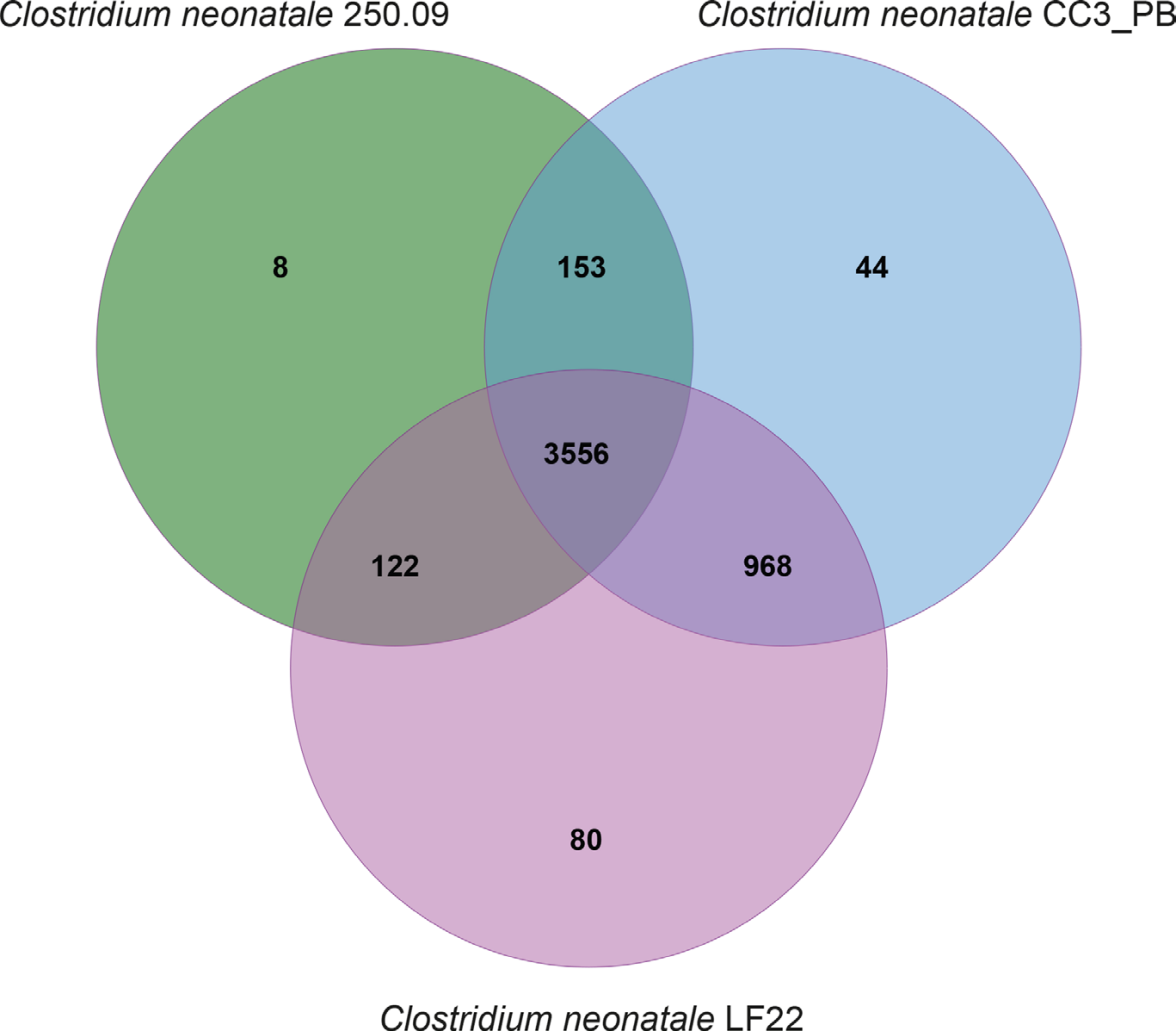
Venn diagram representing the number of shared and unique orthologous protein clusters encoded by the *C. neonatale* 250.09, CC3_PB and LF22 genomes.

A focused orthology analysis of the reference strain 250.09 genome and CC3_PB and LF22 strains having the largest genomes showed 3556 shared protein clusters and 1243 shared by at least two genomes (Fig. 6). Of the 132 unique protein clusters identified, 75 % had neither a Swiss protein ID nor a gene orthologue annotation. Eight unique protein clusters were found in the 250.09 genome, 44 in the CC3_PB genome and 80 in the LF22 genome (Fig. 6, Table S2). In the 250.09 genome, one unique cluster was annotated and associated with virulence and secreted proteins. In the LF22 genome, nine unique protein clusters were identified, including functions related to sporulation, viral genome ejection through the host cell envelope and contractile tail mechanism, cysteine-type peptidase activity, SOS response, ubiquinone biosynthetic process and DNA restriction–modification systems (Table S2). The CC3_PB genome had 11 unique protein clusters identified with functions related to conjugation, single fertilization, polysaccharide metabolic process and transmembrane transport (Table S2).

### Potential mobile genetic elements in *C. neonatale* genomes

A total of 201 potential GIs were predicted (Table 2). They were characterized by a different G+C content compared to the rest of the genome and by the presence of transposition-, integration- and recombination-associated genes (Table S3). The number of predicted GIs ranged from 18 to 32 and had a similar average length (Table 2, Fig. S1). One consensus GI (GI17) was identified and seven (GI3, GI5, GI8, GI10, GI17, GI25 and GI28) were shared among NEC25, NEC26, NEC86, NEC32, C25 and CB12 genomes (Table S3). Strains 250.09, CC3_PB and LF22 had 11, 16 and 19 specific GIs, respectively (Table S3). The ratios of the total size of GIs to the corresponding genome were between 6 and 9 %. Strains LF22 and CC3_PB had the largest total size of GIs, 525 and 429 kb respectively, representing 9 and 7 % of their total genomes. Compared to the 250.09 genome size, the total length of GIs of strains CC3_PB and LF22 represented an increase of 1 % (70 kb) and 3 % (161 kb) in their genome sizes, respectively. Genes identified in GIs coding hypothetical proteins with unknown functions ranged from 22 to 55 %: strains CC3_PB and LF22 had the highest percentages, 55 and 50% respectively.

**Table 2.** Characteristics of *C. neonatale* genomic islands

	250.09	CC3_PB	LF22	CB12	C25	NEC32	NEC26	NEC86	NEC25
No. of GIs	21	25	32	18	22	21	22	19	21
GI total length (kb)	359	429	520	312	345	337	378	290	361
Largest GI (kb)	70	36	41	51	52	52	63	55	52
GI mean length (kb)	17	17	16	16	18	16	17	20	17

Only GIs >5 kb were considered.

GI, genomic island.

Thirteen different IS family members were identified among genomes. They are associated with copy-paste, cut-paste, co-integration, or peel and paste mechanisms of action. On average, genomes contained 8.5±1.7 IS families. The LF22 genome had the highest IS family number (*n*=12) and NEC25 the lowest (*n*=5) (Table S4). Some IS families were found to be absent or shared between genomes while others were unique (Table S4). The ISs had blast matches mostly with *Clostridium* species and other gram-positive bacteria.

One putative CRISPR-Cas Type I-B system (cas6-cas8b-cas7-cas5-cas3-cas4-cas1-cas2) was identified in six (66 %) genomes (Table S5). Five distinct repeats were either shared (*n*=2) or specific (*n*=3) (Table S5). The genomes of strains 250.09, CC3_PB and LF22 had higher spacer numbers (from 11 to 25) compared to the NEC86, NEC32, NEC26 and NEC25 genomes (from four to eight). No putative CRISPR elements were predicted in the CB12 genome.

Intact bacteriophage signatures (score>100) were predicted in the CC3_PB (*n*=7), LF22 (*n*=5), 250.09 (*n*=2) and CB12 (*n*=1) genomes (Table S6). Prophage size lengths ranged between 16 and 70 kb and G+C content varied from 27 and 30 %. Total phage lengths in CC3_PB, LF22, 250.09, and CB12 genomes were 219, 259, 83 and 46 kb, respectively. The proportion of genes coding hypothetical proteins with unknown functions ranged from 26 % (250.09) to 66 % (CB12). Of the 15 intact bacteriophage signatures, nine (60 %) were related to the most common phages of *Clostridium* species (Table S6). Of the seven predicted bacteriophage in the CC3_PB genome, four (57%) were related to *C. difficile* while in the LF22 genome three (60 %) out of five were related to *C. tetani* (Table S6). One phage, phiCT19406C, was shared among the CC3_PB, LF22 and CB12 genomes (Table S6). Some of the identified GIs were included within the predicted prophage signatures, representing 12 % (*n*=4 GIs), 23 % (*n*=5 GIs) and 58 % (*n*=1 GI) of the prophage lengths of LF22, CC3_PB, and CB12, respectively.

Potential elements associated with plasmid sequences were identified in the CC3_PB*,* CB12 and LF22 genomes (Table S7). However, we did not identify specific plasmid replicons or perform a plasmid *de novo* assembly.

### Virulome and resistome

Different potential virulence homologous genes were identified and shared among *C. neonatale* genomes. Genes encoding non-specific virulence factors included flagellar (*flg*), filament formation (*fli*), chemotaxis (*chew*, *mot*)*,* magnesium and iron transport, and ABC transporters (Table S8). ATP-dependent protease (*clp*), conjugated bile salt hydrolase (*cbsh*) and peptide methionine sulphoxide reductase (*msr*), and genes associated with virulence under stressful conditions were found in all genomes (Table S8). Factors involved in adherence (type IV pilus, *pil*) or antiphagocytosis (cell wall modification or capsule production) were also present (Table S8). Most of these genes were located on GIs.

In terms of resistome, the LF22 genome showed the presence of a *tet*(W/N/W) gene encoding a tetracycline resistance ribosomal protection protein (ARO:3004442, 70 % protein sequence identity) (Table S9). This gene was located in GI80, which is unique to LF22 (Table S3). Antimicrobial susceptibility testing confirmed the resistance phenotype of LF22 to tetracycline (MIC=12 mg l^−1^) (Table S10). In the NEC86 genome, the resistance gene encoding a 23S rRNA methyltransferase *ermQ* was identified (ARO:3000593, 100 % protein sequence identity) (Table S9). Strains 250.09, CC3_PB and CB12 were susceptible to all tested antibiotics (Table S10).

## Discussion

In this study, we describe the genomic features and diversity of *C. neonatale *, a newly described and validated species. Taking advantage of the PacBio long sequence reads and shorter HiSeq sequencing data hybrid assembly, we report the closed genome of the *C. neonatale* reference strain 250.09. We also provide a manually curated and re-annotated genome resulting in a better functional precision of genes and addition of new genes compared to the currently available automatic annotation of 250.09 genome scaffolds [9, 19]. In the present study, we also generated new draft genomes for three *C *. *neonatale* clinical isolates (CB12, CC3_PB and LF22) and included five publicly available genomes to perform a comparative genomic analysis. The ANI comparisons revealed a higher genomic variability of our *C. neonatale* genomes as compared to the publicly available genomes. Moreover, comparison of the whole-genome circular maps highlighted the presence of multiple gaps, supporting genome plasticity.

The pan-genome analysis was performed to provide (i) a core-genome containing genes present in all strains, (ii) an accessory genome, which contains genes present in two or more strains, and (iii) unique genes that are specific to single strains. Additionally, pan–core analysis allows the tracing of horizontal gene-flux across strains and provides insight into species evolution [27, 39]. We found the *C. neonatale* genome to be an open pan-genome, indicating the possibility of the addition of gene sets with introduction of new genomes. This result is similar to previous studies on members of the genus *Clostridium sensu stricto* [39]. The core genes code for the basic aspects of the biology of the species and its major phenotypic traits. The highest number of annotated genes in the *C. neonatale* core-genome was related to COG categories previously reported for members of the genus *Clostridium sensu stricto* [39]. However, we found that 79 % of the *C. neonatale* core genes were annotated with COG categories compared to 62 % for members of this genus [39]. Additionally, by comparison, core genes were enriched in categories belonging to ‘cell wall/membrane/envelope biogenesis’, ‘cell motility’, ‘signal transduction mechanisms’, ‘defence mechanisms’, ‘transcription’, ‘energy production and conversion’, ‘carbohydrate transport and metabolism’, ‘amino acid transport and metabolism’, and ‘inorganic ion transport and metabolism’. We confirmed that the *C. neonatale* genome contains dedicated genes for central metabolism including the fermentative pathways of pyruvate into butyrate, ethanol, butanol, lactate and acetate as reported for members of the genus *Clostridium sensu stricto* [40, 41]. Interestingly, genes allowing butanediol production and nitrogen fixation were also found. These are metabolic features that are not common to all *Clostridium* species. In particular, butanediol is a chemical building block of industrial interest with a wide range of potential utilization whose synthesis is based on fossil oil chemical routes. *C. neonatale* may represent a new bacterium with potential metabolic capabilities for biotechnological production of butanediol [42]. Of note, flagella structural genes as well as filament formation and chemotaxis genes were part of the core-genome. We also noted that specific metal detoxification systems such as arsenic (*arsDACB*) and cobalt (*cbiMNQ*) transporters were found in all genomes. These systems contribute to cell homeostasis maintenance in unfavourable environments and may provide *C. neonatale* strains with an ecological advantage in the gut [43]. Interestingly, these genes could be partially, totally or not located in GIs depending on the strain.

Concerning the accessory and unique genes, they usually include supplementary biochemical pathways and functions that may confer selective advantages in terms of ecological adaptation, virulence mechanisms, antibiotic resistance or colonization [27]. In the newly sequenced *C. neonatale* CB12, LF22 and CC3_PB genomes, we found a higher number of unique genes (113 to 742) compared to the other genomes (five to 75 unique genes). These suggest a higher genomic plasticity of these strains that may participate in the observed open pan-genome state of the *C. neonatale* genome. Indeed, the pan-genome ‘open’ state indicate the faculty of new genes acqusition from external environments, thus our data support the ability for *C. neonatale* to acquire exogenous DNA [44, 45]. We also found that recombination and repair categories were related to the accessory and unique genes and were located on GIs or phage signatures, supporting the potential genomic plasticity of *C. neonatale *. Additionally, transport/metabolism categories were enriched in the accessory family genes, suggesting potential homeostasis adaptation of *C. neonatale* to its environment. Cell wall/membrane/envelope biogenesis categories were related to unique gene families, suggesting differences in cellular integrity, protection or pathogenicity among strains. Together, these results support the view of *C. neonatale* with strain genome variability. Although we carried out an analysis of the identified unique genes, we could not clearly highlight particular roles of these genes in the studied genomes. One possible explanation is the high percentage of genes with unknown function distributed among the core, accessory and unique genes despite gene curation and manual re-annotation of the reference genome. Of note, 8 % of the total number of the genes with unknown function were located on GIs and potential prophages. An in-depth analysis of the unique genes is currently in progress.

In strains CC3_PB, LF22 and CB12, multiple potential mobile genetic elements such as GIs, ISs and phages were identified. The presence of a higher number of GIs characterized by a larger total size in these strain genomes suggest more frequent genetic recombination events, which is also reflected in the higher numbers of ISs and intact prophage numbers present in the genomes. When compared against the genome size of strain 250.09, the total length of prophage signatures and GIs accounted for 4 % (648 kb) and 7 % (779 kb) increases of the CC3_PB and LF22 genomes, respectively. These results contribute to the higher genome size of strains CC3_PB and LF22. Concerning prophages, whether they are active and able to excise, leading to cell lethality, needs to be determined. In addition, although we identified potential plasmid-related sequences as a first step, we could not perform a plasmid *de novo* assembly. In-depth analysis of plasmid content using PlasClass complementary bioinformatics tools are ongoing.

Bacteria defend against prophages, plasmids and other foreign DNA mainly through the use of restriction-methylation and CRISPR-Cas systems [46]. Of the nine genomes studied, six had the Type I-B system and different types of CRISPR repeats and spacer contents. The strain CC3_PB and LF22 genomes were characterized by a higher number of spacers, which has been associated with greater genetic diversity of infecting phages [47]. Together, the identification of multiple mobile elements carrying unique and a large number of unknown function genes associated with different defensive mechanisms against foreign DNA support the horizontal gene transfer, genome organization differences and plasticity of the *C. neonatale* genome.

*C. neonatale* virulence determinants remain to be resolved. Here, we identified homologous primary virulence classes including offensive, defensive, and regulation of virulence-associated and non-specific virulence factors. However, because the overall sequence identities were between 40 and 68 %, these data need to be taken with caution and need experimental validation. It could be assumed that variation in the number of GIs and their gene content would contribute to differences in virulence among strains. Although we observed some diversity in terms of unique genes, GIs, and other mobile genetic elements between strains, we could not specifically associate these differences with virulence. Hence, differences in virulence may be attributable to the expression of genes not directly related to virulence, such as loss of genes preventing expression of virulence genes or unknown genes directly or indirectly associated with virulence. In the present study, the low number of strains isolated from infants with or without NEC included in our analysis did not allow us to identify known specific virulence factors as compared to the high percentage of identified unique genes with unknown function. Taking into consideration the open state of the *C. neonatale* genome, a larger genomic comparative analysis is needed as well as experimental studies.

In the present study, we confirmed that the *C. neonatale* 250.09 reference strain was susceptible to previously tested antibiotics [9]. However, we report that strain LF22 was resistant (MIC=12 mg l^−1^) to tetracycline: this strain carries a *tet* gene located in a specific GI and encoding a ribosomal protection protein. Additionally, in strain NEC86, we identified *in silico* a gene encoding a 23S rRNA methyltransferase *ermQ* gene described to provide resistance to macrolides. NEC treatment includes the use of broad-spectrum antibiotics, among which are clindamycin or metronidazole that target anaerobic bacteria [48]. Although one study has reported a significant association between *C. butyricum *-linked NEC and antibiotic administration [48], there are no data regarding the antimicrobial susceptibility of *C. neonatale* clinical isolates. Our data support the need, in future studies, to evaluate *C. neonatale* susceptibility to antibiotics.

## Conclusion

In this study, we present the whole genome assembly of the *C. neonatale* type strain and provide an accurate and up-to-date closed-genome manually validated and curated. The *C. neonatale* pan-genome is considered to be in an open state, genetically diverse, and with a flexible gene repertoire associated with multiple mobile genetic elements. By describing the biological capabilities, plasticity and defence mechanisms of the *C. neonatale* genome, the present study contributes to the understanding of this microorganism and provides a new genetic framework for future studies. We highlight the need for further extending the number of sequenced genomes of this recently described species associated with NEC in preterm neonates, particularly to help elucidate the pathogenic potential of this species.

## Supplementary Data

Supplementary material 1
